# Lingual schwannoma in an adolescent boy: A case report

**DOI:** 10.1016/j.amsu.2021.102216

**Published:** 2021-03-17

**Authors:** Fayez A. Alrohaimi, Sahar A. Alsadah, Ghadeer A. Althaqib

**Affiliations:** a Otolaryngology Head and Neck Surgery, Prince Sultan Military Medical City, Riyadh, Saudi Arabia; b Princess Nourah Bint Abdul Rahman University, College of Medicine, Riyadh, Saudi Arabia

**Keywords:** Schwannoma, Case report, Tongue mass, Adolescent, Transoral-excision

## Abstract

Oral cavity schwannoma is still considered a rare entity in the head and neck region. Schwannoma is a benign encapsulated nerve sheath tumour that originate from Schwann cells with an approximate incidence of 1% in the oral cavity. The tongue is the most common location among oral cavity tumours. We report a rare case of tongue schwannoma in 12-year-old boy who presented to our centre complaining of swelling of the tongue, which had increased in size and interfered with his speech in 2018; he had been previously asymptomatic since birth. A diagnosis was established based on the following imaging studies: computed tomography (CT) and magnetic resonance imaging (MRI). He underwent simple transoral excision of the ventral tongue mass under general anaesthesia with no complications, and the final pathological result confirmed the diagnosis of schwannoma.

## Highlights

• Oral cavity schwannoma is a rare tumour in the head and neck region.
• CT and MRI help in the diagnosis.
• Transoral excision is the most common procedure.
• Pathological examination confirms the diagnosis.

## 1 Introduction

Throughout history, from 1945 when Pignatelli reported a case of a young male with a diagnosis of lingual schwannoma until now, 2020, oral cavity schwannoma is still considered a rare entity [1]. Schwannoma or neurilemmoma is a benign encapsulated nerve sheath tumour that originates from Schwann cells and is predominantly locate in the head and neck area [2]. Approximately 25–40% of cases occur in the head and neck region mostly in the parapharyngeal space with an incidence of only 1% in the oral cavity [3,4]; among subsites in the oral cavity, the tongue is the most common location [5].

## 2 Case report

Has been reported in line with the SCARE 2018 criteria.

A 12-year-old medically free boy presented to the Head and Neck Clinical Department of Prince Sultan Military Medical City (PSMMC) in Riyadh in 2016 complaining of tongue swelling “at the age of 8 year old” and who had been asymptomatic since birth; , the tongue had not increased in size or changed colour, there was no history of pain, burning sensation, dysphagia, paraesthesia, bleeding from the lesion. There were no other masses in his mouth or other body areas. There was no history of allergies with negative family history of any head and neck masses or genetic disease. On examination, a smooth cystic mass was observed on the right side of the ventral tongue, with no other abnormalities detected in the nose, nasopharynx, larynx, ear or neck.

**Fig. 1:**
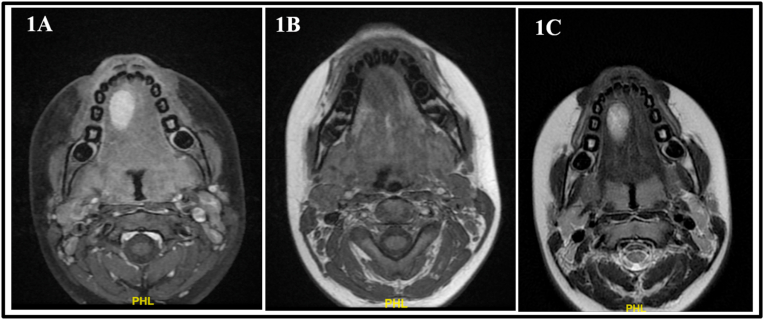
**Includes 1A, 1B, 1C** and shows the MRI findings demonstrating a well-defined lesion measuring 2.3 × 1.4 × 2.0 cm in size in AP, transverse and craniocaudal dimensions, respectively; note that the lesion is embedded within the intrinsic muscle of the oral tongue on the right side smoothly indenting the genioglossus/geniohyoid muscle complex, which is minimally displaced to the left side. Figure 1A: Axial T1-weighted image with contrast showing avid enhancement on post contrast images with no evidence of diffusion restriction. Fig. 1B: Axial T1-weighted image show the hypointense lesion**.**Fig. 1**C:** Axial T2-weighted image show heterogenous hyperintensity.

**Fig. 2:**
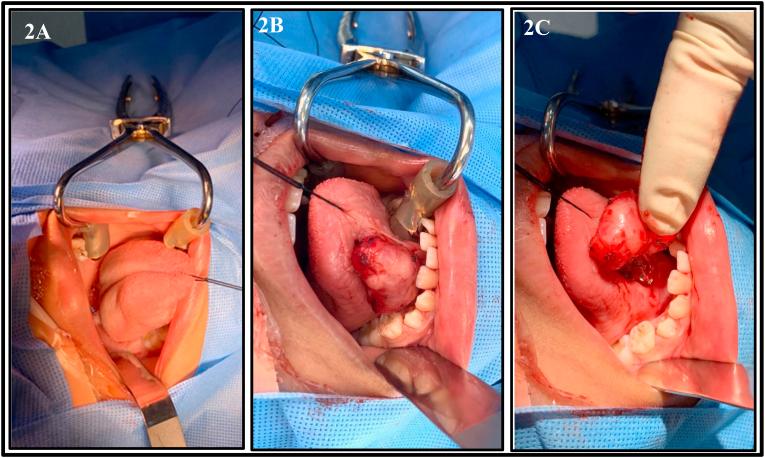
Shows the intraoral surgical excision after draping with chlorhexidine mouthwash. Fig. 2**A:** 3–0 retraction suture placed at the tip of the tongue for better exposure. Fig. 2B: incision and dissection were performed. Fig. 2C: cyst delivered completely and sent for histopathology.

In 2018 the tongue mass started to increase in size and interfere with speech, but laboratory investigations were normal. The patient underwent a contrast-enhanced CT scan, which was inconclusive, and the MRI with infusion results are show in Fig. 1. The patient was admitted in December 2018 and underwent intra-oral excision of the ventral tongue mass under general anaesthesia by head and neck consultant with no complications **(**Fig. 2**)**. The patient was discharged on the same day with antibiotics and analgesia, with instruction to consume a soft diet before advancing, and with follow-up after 2 weeks. The patient currently undergoing follow-up in the clinic; since discharge, there have been no signs or symptoms of recurrence.

## 3 Differential diagnosis

• Benign capillary haemangioma
• Venolymphatic malformation
• Retention cyst ranula

## 4 Histopathology

**Grossly:** round to oval mass measuring 2.3 × 2 cm in size. The outer surface was smooth.

**Microscopically:** Serial sectioning revealed a tan-white cassette strongly positive for S100, focally positive for CD56 and negative for NFR.

Final pathological diagnosis: schwannoma.

## 5 Discussion

In 1908, Jose Verocay made the first description of the benign tumour known currently as schwannoma [6]. Though it is a benign tumour, the malignant transformation rate is reported to be between 8% and 13.9%, while the lingual schwannoma transformation rate is 2.8% [[7], [8], [9]]. Clinically, schwannoma can appear at any age with no sex predilection and with a variety of manifestations depending on its size and location; in most cases, it presents as a smooth, well-defined, asymptomatic mass. Pain, dysarthria, dysphagia snoring, and sleep apnoea, have been reported [[10], [11], [12]]. Likewise, our patient presented with: smooth cystic mass, which was asymptomatic at the beginning and then started to interfere with his speech. The differential diagnoses included but were not limited to salivary gland tumours, lymphangiomas, haemangiomas, epidermoid cysts, lipomas, inflammatory lesions and, schwannomas [13]. Imaging and histopathological finding were used to differentiate between these tumours.

The usual CT findings are a well-defined mass with heterogeneous enhancement with possible cystic or fatty degeneration, and these finding were inconclusive in our case [14]. The MRI characteristics were a hypointense lesion on T1-weighted imaging, heterogenous hyperintensity on T2-weighted imaging, and avid enhancement on post-contrast imaging, with no evidence of diffusion restriction on T1-weighted imaging, which contrasted with the findings reported in the literature. In addition, this mass was strongly positive for S100 and focally positive for CD56, which is typically defined in these tumours. Antoni A and B were arranged in palisading patterns and were composed of hyper- and hypocellular areas, respectively [15]. The definitive management of intraoral schwannoma is surgical excision via a variety of approaches depending on the size and location of the tumour, ranging from simple transoral excision, as was used in this case, to the more complex open approach.

## 6 Conclusion

Tongue schwannoma is a comparatively rare tumour. The clinical presentation ranges from a totally asymptomatic mass to a mass that is rapidly increasing in size and causes dysarthria, dysphagia snoring, sleep apnea and pain. Rarely, lingual schwannoma transformation occurs. Pathological examination confirms the diagnosis. Transoral excision is the most common procedure, but a variety of approaches have been established depending on the size and location of the tumour.

## Provenance and peer review

Not commissioned, externally peer-reviewed.

## Source of funding

No funding.

## Ethical approval

Nil

## Consent

Written informed consent was provide by the patient and his parents for publication purposes, with full awareness of the use of his images and information.

## Author contribution

Fayez A Alrohaimi, Sahar A Alsadah, Ghadeer A Althaqib all contribute in the study concept and design, writing the paper.

## Registration of research Studies

1. Name of the registry:
2. Unique Identifying number or registration ID:
3. Hyperlink to your specific registration (must be publicly accessible and will be checked):

## Guarantor

Sahar A Alsadah

## Declaration of competing interest

The authors declare that there are no conflicts of interest.
